# Knowledge, Attitudes, and Perceptions of the Physician’s Role in Colorectal Cancer Screening Among Primary Care Patients in Msaken, Tunisia, 2026

**DOI:** 10.5888/pcd23.260083

**Published:** 2026-09-24

**Authors:** Rania Bannour, Lassaad Trabelssi, Omar Khalil Ben Saad, Amani Hassine, Jihene Sahli, Amara Amel, Mariem Ghardallou, Manel Mallouli, Chekib Zedini

**Affiliations:** 1 University of Sousse, Faculty of Medicine of Sousse, Sousse, Tunisia; 2 Primary Health Group, Sousse, Tunisia; 3 Department of Hepato-gastroenterology, Ibn al Jazzar University Hospital, Kairouan, Tunisia

## Abstract

**Introduction:**

Colorectal cancer (CRC) is a major public health concern worldwide and an emerging health issue in Tunisia. Early screening is critical for prevention and early diagnosis. Our study aimed to assess knowledge, attitudes, and behaviors related to CRC screening among primary care patients and to examine factors associated with screening acceptance, including the perceived role of physicians, as well as the barriers to discussing CRC.

**Methods:**

We conducted a cross-sectional study among adults consulting primary health care centers in Msaken, Tunisia, in January 2026. Data were collected by using a validated, structured questionnaire that assessed sociodemographic characteristics, knowledge of CRC and screening methods, sources of information, physician–patient communication, attitudes toward screening, and perceived barriers. Associations were analyzed by using univariate and multivariate logistic regression.

**Results:**

The study sample consisted of 701 participants. Among them, 72.0% had heard of CRC, but only 5.8% correctly identified the recommended age for screening. Almost one-third (32.8%) reported awareness of the hemoccult test, and 64.6% expressed willingness to undergo the test if it was free. We identified several independently associated factors with acceptance of a hemoccult test, including prior awareness of CRC (odds ratio [OR] = 2.18; *P* < .001), family history of CRC (OR = 2.00; *P* < .001), exposure to medical internet sources (OR = 2.06; *P* < .001), and prior discussion with a physician (OR = 2.46; *P* < .001).

**Conclusion:**

Although patient attitudes were generally positive, we found notable gaps in knowledge and screening practices. The strongest determinant of screening acceptance was doctor–patient communication, which shows the importance of the role primary care physicians in CRC prevention.

**Summary**

**What is already known on this topic?**

Colorectal cancer (CRC) screening rates are low in many middle‑income countries, and physician recommendation is a key driver of screening behavior.

**What is added by this report?**

A cross‑sectional study of 701 primary care patients in Msaken, Tunisia, demonstrated gaps in patient knowledge of CRC. Physician–patient discussions strongly increased acceptance of fecal occult blood testing.

**What are the implications for public health practice?**

Strengthening physician communication skills and incorporating systematic screening counseling in primary care could improve early detection and reduce CRC prevalence in Tunisia and similar settings.

## Introduction

Colorectal cancer (CRC) is one of the most common cancers worldwide. It is the third most commonly diagnosed cancer and the second leading cause of cancer death globally, according to the World Health Organization (1). In 2020, there were an estimated 1.9 million new cases and nearly 935,000 deaths from this cancer worldwide (1). 

Most cases of CRC occur after the age of 50 years (2). However, cases among younger people are increasing in several countries. Early detection would allow for the identification of precancerous lesions or cancers at a curable stage, significantly reducing mortality rates. Simple methods, such as the fecal immunochemical test or the fecal occult blood test, are recommended as tools for first-line screening (3).

Despite the proven effectiveness of screening, its coverage remains low in many low- and middle-income countries due to a lack of information, stigma, cultural taboos, or insufficient access to care. In Tunisia, CRC is the second most common cancer in men and the third in women, according to regional registries. Its incidence is increasing, particularly in coastal regions and semi-urban areas (4). However, the national CRC screening program remains poorly structured, and no systematic nationwide campaign exists.

Tunisia benefits from a robust primary health care network (5). However, studies on the role of the physician in CRC prevention are scarce. In a cultural context in which talking about the colon, stool, or rectum is perceived as embarrassing, assessing patients’ perceptions of their doctors, the barriers related to embarrassment or taboos, and their level of information about available tests is crucial (6).

Primary care physicians play a central role in promoting CRC screening. As the first point of contact with the health care system, they are ideally positioned to inform, raise awareness among, and motivate eligible patients to undergo screening tests (7). Their active involvement in health education not only helps to correct misconceptions and break down taboos but also strengthens patient adherence to screening recommendations. A trusting doctor–patient relationship is essential to fostering open dialogue on CRC issues, particularly in sociocultural contexts in which these topics may be perceived as sensitive (8).

This study aimed to assess the knowledge, attitudes, and practices of primary care patients in the Tunisian city of Msaken about CRC and CRC screening, to explore the perceived role of the physician in screening education, and to identify barriers to discussing CRC with health care professionals.

## Methods

### Type of study

A cross-sectional study was conducted from January 2 through January 31, 2026, among adult patients (≥18 years) who consulted primary health care centers in Msaken for reasons other than known cancer. Eligible participants were those without a personal history of CRC or chronic inflammatory bowel disease. Patients who had undergone a colonoscopy within the past year were also excluded. Medical and paramedical staff were not included in the study. All participants provided informed consent prior to inclusion.

### Data collection

Data were collected by using a validated, structured questionnaire administered in Arabic. The tool was self-administered with the assistance of a trained interviewer when needed to ensure comprehension of items and to minimize bias related to educational level.

The questionnaire was developed following a comprehensive review of the international literature, based on validated instruments that assessed knowledge, attitudes, and practices about CRC screening (6,9–11). A pilot test was conducted on a small sample of the target population to assess clarity, feasibility, and comprehensibility. Necessary modifications were made prior to final deployment. Participants were recruited on a voluntary basis from primary health care facilities and met the predefined inclusion criteria. After receiving a brief explanation of the study objectives and providing informed consent, eligible participants completed the self-administered questionnaire, with assistance when needed. 

The questionnaire assessed sociodemographic characteristics (sex, age, education level, marital status), family history of CRC, knowledge of CRC and its clinical symptoms, knowledge of screening methods, sources of CRC information, perceived role of the physician, and barriers to discussing CRC screening. 

### Sample size and sampling method

A nonprobability convenience sampling strategy was used. The minimum required sample size was estimated by using the Schwartz formula based on an assumed 50% prevalence of adequate knowledge, a 95% confidence level, and a 5% margin of error; therefore, the minimum sample was 385 participants. All eligible participants who agreed to take part in the study during the data collection period were included, resulting in a final sample of 701 participants.

### Analytical methods

Statistical analysis was performed by using SPSS version 24 (IBM Corporation). A χ^2^ test was performed for categorical variables in independent samples, and categorical variables were calculated as percentages. The outcome-dependent variable was acceptance (yes or no) of a hemoccult test. Multivariate logistic regression was used to assess the association between acceptance of a hemoccult test and the independent variables. Model fit and assumptions were assessed by using the Hosmer–Lemeshow goodness-of-fit test, the Cox and Snell and Nagelkerke pseudo *R* ^2^ likelihood ratio tests, and log-likelihood values. Odds ratios (ORs) with 95% CIs were calculated. A 2-tailed *P* value of less than .05 was considered significant, and variables with a *P* value of less than .20 were included in the model.

### Ethical considerations

The study was conducted in accordance with the ethical principles of biomedical research. Informed consent was obtained from all participants prior to inclusion. Participation was voluntary, and participants were informed of their right to refuse or withdraw at any time without consequence. Anonymity and confidentiality were strictly ensured. No identifying personal information was collected, and all data were handled in a confidential manner and used solely for research purposes. Ethical approval for this study was obtained from the Ethics Committee of the University Hospital Sahloul Sousse (approval no. HS 44-2025).

## Results

Most participants were female (63.1%). Of the total sample, 45.4% of participants were aged 20 to 39 years, and 24.1% were aged 50 years or older, the age considered to be at high risk for CRC. Most participants were moderately to highly educated; 34.0% had a secondary education, and 29.2% had a university education. More than half of participants were married (57.8%). Overall, 9.8% of participants reported a family history of CRC (Table 1).

**Table 1:** Sociodemographic Characteristics of Participants (N = 701), Survey on Knowledge, Attitudes, and Perceived Role of the Physician in Colorectal Cancer Screening, Msaken, Tunisia, 2026

Variable	No. (%)
**Sex**	
Male	259 (36.9)
Female	442 (63.1)
**Age, y**	
<20	67 (9.6)
20–29	163 (23.3)
30–39	155 (22.1)
40–49	147 (21.0)
≥50	169 (24.1)
**Level of education**	
Primary	112 (16.0)
Secondary	238 (34.0)
College	108 (15.4)
University	205 (29.2)
Postgraduate	38 (5.4)
**Marital status**	
Single	240 (34.2)
Married	405 (57.8)
Divorced or widowed	56 (8.0)
**Family history of colorectal cancer**	
Yes	69 (9.8)
No	559 (79.7)
Doesn’t know	73 (10.4)

### Knowledge level, information sources related to CRC and associated risk factors, and perceptions of the physician’s role

Overall, 72.0% of participants had heard of CRC; however, notable gaps in anatomical and functional knowledge persisted; only 66.5% correctly identified the colon as part of the digestive system, and only 46.5% recognized its physiological role (Table 2). Awareness of CRC frequency and risk factors was heterogeneous: fewer than half of participants (39.1%) perceived CRC as a common disease, 27.7% were unable to identify any risk factor, and only 3.9% correctly recognized all listed risk factors. Lifestyle-related factors — including unhealthy diet, smoking, alcohol consumption, sedentariness, stress, and obesity — were widely acknowledged, whereas diabetes and sex were less frequently cited. Knowledge of clinical warning signs was limited, with only 10.4% correctly identifying all 4 key symptoms and more than one-third recognizing none; chronic abdominal pain was the most frequently reported symptom (45.1%), while unexplained weight loss was the least recognized (29.8%). Less than half of participants (47.5%) correctly identified colonoscopy with biopsy as the confirmatory diagnostic test (Table 2).

**Table 2:** Information Sources Related to CRC, Knowledge of CRC and Its Risk Factors, and Perceptions of the Physician’s Role in CRC Screening, Participants (N = 701) in a Survey on Knowledge, Attitudes, and Perceived Role of the Physician in CRC Screening, Msaken, Tunisia, 2026

Topic	No. (%)
**Have you ever heard of CRC?**	
Yes	505 (72.0)
No	196 (28.0)
**Do you know what the colon is?**	
Part of the digestive system (correct answer)	466 (66.5)
Gland	9 (1.3)
Don’t know	226 (32.2)
**Do you know the function of the colon?**	
Water absorption and fecal matter formation (correct answer)	326 (46.5)
Enzyme synthesis	21 (3.0)
Don’t know	354 (50.5)
**Is CRC a common disease?**	
Yes	274 (39.1)
No	92 (13.1)
Don’t know	335 (47.8)
**What are the risk factors of CRC?^a^**	
Sex	396 (56.5)
Family history of CRC	335 (47.8)
Diet rich in industrially produced meat	410 (58.5)
Alcoholism	401 (57.2)
Smoking	420 (59.9)
Stress	401 (57.2)
Sedentary lifestyle	391 (55.8)
Diet high in saturated fats	363 (51.8)
Obesity or overweight	373 (53.2)
Low-fiber diet	342 (48.8)
Low-vegetable diet	342 (48.8)
Diet rich in red meat	260 (37.1)
Low-fruit diet	256 (36.5)
Advanced age	257 (36.7)
Diabetes	159 (22.7)
**What are the clinical signs of colorectal cancer?**	
**Rectal bleeding or presence of blood in the stool**	
Yes	258 (36.8)
No	443 (63.2)
**Alternating diarrhea and constipation**	
Yes	263 (37.5)
No	438 (62.5)
**Chronic abdominal pain**	
Yes	316 (45.1)
No	385 (54.9)
**Unexplained weight loss**	
Yes	209 (29.8)
No	492 (70.2)
**What is the diagnostic confirmation test for CRC?**	
Colonoscopy plus biopsy	333 (47.5)
Scanner	84 (12.0)
Urine test	34 (4.9)
Don’t know	250 (35.7)
**What are your sources of information about CRC?^a^**	
Social media	249 (35.5)
Medical internet sources	184 (26.2)
Physician or nurses	176 (25.1)
Family and friend	210 (30.0)
Radio or television	133 (19.0)
**Physician–patient discussion**	
**Did you ever discuss CRC with your physician?**	
No	513 (73.2)
Yes	104 (14.8)
Rarely	84 (12.0)
**In your opinion, physician should explain screening importance**	
Yes	689 (98.3)
No	12 (1.7)
**Do you feel any barriers when discussing CRC with your physician?**	
Didn’t consider the subject before	363 (51.8)
No	133 (19.0)
Yes	205 (29.2)
**What are the barriers to discussing screening with doctor?^b^**	
Embarrassment/shame	108 (52.7)
Insufficient consultation time	97 (47.3)

Abbreviation: CRC, colorectal cancer.

a Participants could choose more than 1 answer.

b Of the 205 participants who said they felt uncomfortable addressing the topic of CRC.

The main sources of CRC-related information were social media (35.5%) and the internet (26.2%), while physicians and nurses were cited by only 25.1% of participants. Despite this, an overwhelming majority (98.3%) believed that physicians should explain the importance of screening and early detection. Nevertheless, 73.2% reported never having discussed CRC with their physician, and only 14.8% indicated they did so. This communication gap was compounded by psychologic and systemic barriers: 29.3% of participants felt uncomfortable addressing the topic; of these participants, 52.7% cited embarrassment or shame, and 47.3% cited insufficient consultation time (Table 2).

### Attitudes, opinions, and behaviors toward CRC screening

Most participants (70.0%) recognized that early detection is a means of preventing CRC. However, nearly a third (30.0%) did not perceive its preventive benefits. Nearly half of participants (48.2%) stated they did not know the age at which regular screening should begin, and only 5.8% correctly answered 50 years of age. Furthermore, awareness of the hemoccult test was limited; only 32.8% had heard of it and only 3.1% had actually had it performed. In terms of perceptions, more than half (50.9%) considered the hemoccult test to be unproblematic, while 20.3% found it embarrassing. Nevertheless, most participants (64.6%) said they would be willing to take the test if it were offered free of charge (Table 3).

**Table 3:** Attitudes, Opinions, and Behaviors Toward Colorectal Cancer Screening, Participants (N = 701) in a Survey on Knowledge, Attitudes, and Perceived Role of the Physician in Colorectal Cancer Screening, Msaken, Tunisia, 2026

Variable	No. (%)
**Is early detection a means of prevention?**	
Yes	491 (70.0)
No	210 (30.0)
**Age at which regular screening begins, y**	
30	152 (21.7)
40	170 (24.3)
50	41 (5.8)
Don’t know	338 (48.2)
**Have you ever heard about the hemoccult test?**	
Yes	230 (32.8)
No	471 (67.2)
**Would you take the hemoccult test?^a^**	
Yes, if offered free of charge	453 (64.6)
Maybe	247 (35.2)

a Information for 1 participant was missing.

### Factors associated with acceptance of a hemoccult test

Acceptance of a hemoccult test did not differ significantly according to sex (*P* = .15) or educational level (Table 4) (*P* = .22). A nonsignificant association was observed between family history of CRC and acceptance of a hemoccult test (*P* = .05), with higher acceptance rates among participants reporting a positive family history.

**Table 4:** Factors Associated With Acceptance of a Hemoccult Test, Participants (N = 701) in a Survey on Knowledge, Attitudes, and Perceived Role of the Physician in CRC Screening, Msaken, Tunisia, 2026

Variable	Acceptance, no. (%)	No acceptance, no. (%)	*P* value
**Sex**			
Male	159 (22.7)	100 (14.3)	.15
Female	295 (42.1)	147 (20.1)	
**Educational level**			
Secondary or less	304 (43.4)	154 (21.9)	.22
University	150 (21.4)	93 (13.4)	
**Family history of CRC**			
No	229 (32.6)	403 (57.5)	.05
Yes	51 (7.3)	18 (2.6)	
**Awareness of CRC**			
Yes	353 (50.3)	152 (21.7)	<.001
No	101 (14.4)	95 (13.6)	
**Source of information**			
**Medical internet sources**			
Yes	140 (30.8)	44 (17.8)	<.001
No	314 (69.2)	203 (82.2)	
**Social media**			
Yes	170 (37.4)	79 (32.0)	.15
No	284 (62.6)	168 (68.0)	
**Physician or nurse**			
Yes	136 (30.0)	40 (16.2)	<.001
No	318 (70.0)	207 (83.8)	
**Family or friends**			
Yes	147 (32.4)	63 (25.5)	.06
No	307 (67.6)	184 (74.5)	
**Television or radio**			
Yes	98 (21.6)	35 (14.2)	.02
No	356 (78.4)	212 (85.8)	
**Role of the physician encouraging CRC screening**			
**Discussion with physician about CRC**			
Yes	82 (18.1)	22 (8.9)	<.001
Rarely	63 (13.9)	21 (8.5)	
No	309 (68.0)	204 (82.6)	
**Physician should explain CRC screening**			
Yes	448 (98.7)	241 (97.6)	.28
No	6 (1.3)	6 (2.4)	

Abbreviation: CRC, colorectal cancer.

Awareness of CRC was strongly associated with acceptance of a hemoccult test. Participants who had previously heard of CRC (50.3%) were significantly more likely to accept a hemoccult test compared with those who were unaware of CRC (14.4%) (*P* < .001).

Acceptance of the hemoccult test was also significantly associated with several informational and communication-related factors. Participants who reported medical internet sources as a source of information were more likely to accept the hemoccult test than those who did not (30.8% vs 17.8%) (*P* < .001). Similarly, receiving information from a physician or a nurse was strongly associated with test acceptance (*P* < .001). Of patients who received information from a physician or nurse, 30.0% accepted the test, whereas 16.2% did not (*P* < .001). Exposure to information via television or radio was likewise positively associated with test acceptance (*P* = .02). By contrast, no significant associations were observed between acceptance of the hemoccult test and information obtained through social media (*P* = .15) or from family and friends (*P* = .06).

Prior discussion with a physician about CRC was significantly associated with higher acceptance of a hemoccult test (18.1% vs 8.9%) (*P* < .001). However, the belief that explaining screening is the physician’s responsibility was not significantly associated with test acceptance (*P* = .28) (Table 4).

### Predictive factors of acceptance of a hemoccult test 

Participants with a family history of CRC (vs no family history) were more likely to accept a hemoccult test (OR = 2.00, *P* < .001), as were those with prior awareness of CRC (vs no prior awareness) (OR = 2.18, *P* < .001). Sources of information also influenced acceptance: people who reported medical internet sources (OR = 2.06, *P* < .001), physicians or nurses (OR = 2.21, *P* < .001), or television or radio (OR = 1.67, *P* = .01) as information sources were more likely to accept a hemoccult test than those who did not use those sources, respectively. Discussion with a physician about CRC strongly increased test acceptance (OR = 2.46, *P* < .001). In contrast, sex, educational level, social media, family or friends as sources, embarrassment, and the belief that physicians should explain screening were not significantly associated with acceptance of a hemoccult test (Table 5).

**Table 5:** Predictive Factors of Acceptance of a Hemoccult Test, Participants (N = 701) in a Survey on Knowledge, Attitudes, and Perceived Role of the Physician in CRC Screening, Msaken, Tunisia, 2026

Variable	OR (95% CI)	*P* value
Sex (female vs male)	1.26 (0.93–1.70)	.15
Education level (university vs secondary)	0.82 (0.61–1.10)	.22
Family history of CRC (yes vs no)	2.00 (1.10–3.65)	<.001
Awareness of CRC (yes vs no)	2.18 (1.60–2.98)	<.001
Source of information		
Medical internet sources (yes vs no)	2.06 (1.41–3.01)	<.001
Social media as a sour (yes vs no)	1.27 (0.93–1.73)	.14
Physician or nurse (yes vs no)	2.21 (1.48–3.31)	<.001
Family or friends (yes vs no)	1.40 (1.00–1.97)	.05
Television or radio (yes vs no)	1.67 (1.12–2.48)	.01
Discussion with physician (yes vs no)	2.46 (1.49–4.06)	<.001
Feeling embarrassed discussing the subject with the physician (yes vs no)	0.95 (0.48–1.86)	.87
Physician should explain (yes vs no)	1.86 (0.58–5.95)	.28

Abbreviation: CRC, colorectal cancer.

## Discussion

This study provides insights into the level of knowledge, attitudes, and behaviors related to CRC screening among primary care patients in a Tunisian city and highlights the perceived role of physicians in promoting screening. Overall, while general awareness of CRC was relatively high, substantial gaps were observed in knowledge of risk factors, warning signs, screening modalities, and recommended screening age. These findings underscore the critical need for strengthened physician–patient communication and structured public health interventions.

In our study, 72.0% of participants had heard of CRC. This level of awareness is comparable to that reported in other middle-income countries, where awareness ranges between 60% and 80% (12,13). However, awareness alone did not translate into accurate biomedical knowledge. Only two-thirds of participants correctly identified the colon as part of the digestive system, and fewer than half recognized its physiologic function. Similar deficiencies have been documented in studies conducted in Morocco, Egypt, and Saudi Arabia, where basic anatomical knowledge of CRC was found to be limited despite moderate overall awareness (14–16).

Knowledge of CRC risk factors was heterogeneous. Although lifestyle-related factors such as smoking, unhealthy diet, obesity, and sedentariness were widely cited, advanced age and diabetes were poorly recognized. This pattern is consistent with patterns in previous studies showing that the public tends to overestimate behavioral risks while underestimating nonmodifiable factors such as age and metabolic diseases (17). Notably, only 3.9% of participants correctly identified all listed risk factors, reflecting fragmented and incomplete knowledge. Comparable findings were reported in a Tunisian study on breast and cervical cancer screening, suggesting a broader gap in cancer-related health literacy (18).

Awareness of CRC warning signs was particularly low, with only 10.4% correctly identifying all major symptoms. Rectal bleeding, a cardinal symptom emphasized in international guidelines, was recognized by only 36.8% of participants. This finding is concerning, as poor symptom recognition is a major contributor to delayed diagnosis and advanced-stage presentation in low- and middle-income countries (19). Studies from Europe and Asia report significantly higher recognition rates of rectal bleeding (60%–80%), highlighting a clear knowledge disparity (20,21).

Although 70.0% of participants acknowledged that early detection is a means of prevention, only 5.8% correctly identified 50 years as the recommended age to begin CRC screening. Nearly half of participants reported not knowing the appropriate screening age. Similar results have been reported in several African and Middle Eastern countries, where knowledge of screening guidelines remains poor even among people who express favorable attitudes toward prevention (22).

Awareness of the hemoccult was limited (32.8%), and only 3.1% had ever undergone the test. These rates are substantially lower than those reported in countries with organized screening programs, such as France or the United Kingdom, where participation rates range from 30% to 60% (23). However, they are comparable to rates observed in other countries lacking structured national screening programs (24). Encouragingly, nearly two-thirds of participants expressed willingness to undergo the test if offered free of charge, suggesting that financial and organizational barriers play a key role in low screening rates.

Social media and the internet were the most frequently cited sources of CRC-related information, while health care professionals were mentioned by only one-quarter of participants. This shift toward digital information sources mirrors global trends (25). However, unlike medical internet sources, social media exposure was not significantly associated with screening acceptance. This finding aligns with the findings of previous studies indicating that unregulated health information on social media may increase awareness without necessarily improving preventive behaviors (26).

In contrast, information received from physicians or nurses was strongly associated with acceptance of the hemoccult test, both in univariate and multivariate analyses (OR = 2.21). Prior discussion with a physician emerged as the strongest predictor of screening acceptance (OR = 2.46). These results are consistent with extensive evidence demonstrating that physician recommendation is among the most powerful determinants of CRC screening use (27–29).

Despite this, nearly three-quarters of participants reported never having discussed CRC with their physician. This communication gap represents a missed opportunity for prevention, particularly within Tunisia’s robust primary health care system. Similar gaps have been observed in other sociocultural contexts in which CRC-related discussions are perceived as sensitive or embarrassing (30).

Multivariate analysis identified family history of CRC, prior awareness of CRC, exposure to medical internet sources, traditional media (television or radio), and physician discussion as independent predictors of screening acceptance. These findings are consistent with the Health Belief Model, which emphasizes perceived susceptibility, perceived benefits, and cues to action as key drivers of preventive behavior (31). The absence of a significant association with educational level suggests that targeted physician communication may help reduce social inequalities in screening use.

### Strengths and limitations

Our study has several strengths. One strength was the large sample size that exceeded the minimum total required, which enhanced its statistical power. The use of a validated, structured questionnaire also allowed for comprehensive assessment of knowledge, attitudes, and behaviors. Additionally, the focus on physician–patient interaction provided valuable insights for primary care–based interventions, an area that remains underexplored in Tunisia.

Several limitations should be acknowledged. The use of convenience sampling limited the generalizability of findings beyond the study area. Self-reported data may be subject to recall and social desirability bias. The cross-sectional design precludes causal inference between identified factors and screening acceptance. Finally, the relatively young age of participants may have underestimated the knowledge and behaviors among the recommended screening population.

### Recommendations

Based on these findings, several actions are recommended:

1. Strengthening physician training in CRC prevention and communication skills, particularly in primary care.
2. Integrating systematic CRC screening counseling into routine consultations for eligible patients.
3. Developing culturally adapted educational campaigns, emphasizing warning signs, screening age, and available tests.
4. Leveraging reliable medical internet platforms and traditional media to disseminate evidence-based information.
5. Implementing a structured national CRC screening program with free or subsidized fecal occult blood testing to improve uptake.

### Conclusion

This study highlights gaps in knowledge and screening practices related to CRC among primary care patients in Msaken, Tunisia, despite generally positive attitudes toward prevention. Physician–patient communication emerged as the most influential factor associated with screening acceptance, outweighing sociodemographic and psychologic barriers. These findings emphasize the pivotal role of primary care physicians in CRC prevention and underscore the urgent need for structured screening programs and tailored educational interventions. Strengthening physician engagement and improving public health literacy could substantially enhance early detection and reduce the prevalence of CRC in Tunisia.
